# Renoprotective Action of Linagliptin Among Diabetic Kidney Disease Patients: A Systematic Review and Meta-Analysis

**DOI:** 10.7759/cureus.81833

**Published:** 2025-04-07

**Authors:** Prem S Panda, Ipsa Mohapatra, Sourav Padhee, Ipsita Debata, Somen K Pradhan, Amita Mukhopadhyay, Tejas J

**Affiliations:** 1 Community Medicine, Kalinga Institute of Medical Sciences, Bhubaneswar, IND; 2 Biostatistics, KPMG India, Mumbai, IND; 3 Community Medicine, Maharaja Krishna Chandra Gajapati (MKCG) Medical College, Berhampur, IND; 4 Community Medicine, Institute of Health Management Research, Bengaluru, IND; 5 Forensic Medicine, Karpaga Vinayaga Institute of Medical Sciences and Research Center, Maduranthagam, IND

**Keywords:** diabetes, dipeptidyl peptidase-4, dpp-4, linagliptin, nephropathy, renoprotective

## Abstract

Despite pharmacological and lifestyle changes, hyperglycemia management in type 2 diabetes mellitus patients, combined with a risk for cardio-renal problems, continues to present a significant challenge for doctors. Linagliptin has been found to have a renoprotective effect in diabetic nephropathy patients in recent studies. This systematic review and meta-analysis (SRMA) was conducted to synthesize available information so as to better understand if linagliptin has a renoprotective effect in type 2 diabetes mellitus patients with nephropathy. Three databases were searched from January 2013 to December 2022 to identify all published studies reporting “linagliptin’s effect in patients with diabetic nephropathy”. Owing to the heterogeneity of the included studies, the authors have employed a random-effects model to examine the data in this meta-analysis. Standardized mean differences (SMD) for continuous outcomes were used to compute effect sizes. The final analysis included five studies. The change in estimated glomerular filtration rate (eGFR) did not show a statistically significant difference between the group on linagliptin and the other group on placebo, at baseline (mean difference = 1.19, p = 0.28), after three months of intervention in the selected three studies (mean difference = 0.51, p = 0.85), and after six months of intervention in the selected two studies (mean difference = 0.72, p = 0.70). The study found that linagliptin did not have a significant renoprotective effect in patients with type 2 diabetes, indicating no substantial benefit in slowing kidney disease progression. For diabetes patients with chronic renal disease, linagliptin may be a helpful renoprotective treatment choice, but in the future, more robust and longer-duration research is warranted to establish the inference.

## Introduction and background

Globally, more than 500 million people are currently suffering from type 2 diabetes mellitus, the main cause of renal and cardiovascular problems [1]. Type 2 diabetes mellitus is a leading cause of diabetic nephropathy (DN), a progressive kidney disease resulting from chronic hyperglycemia-induced damage to the renal microvasculature. Managing hyperglycemia in these patients who are at risk for cardiovascular and renal problems is still a significant challenge for doctors who treat this population, even with lifestyle and medication changes [2,3]. DN is still a serious complication related to type 2 diabetes mellitus, culminating in more severe conditions like chronic kidney disease (CKD) and end-stage renal disease (ESRD), associated with a decreased quality of life, morbidity, and death. About 35% of type 2 diabetes mellitus have DN, leading to ESRD, the most prevalent reason for microvascular complications and mortality related to cardiovascular disease [4]. Diabetic individuals who receive hemodialysis are known to experience more difficulties compared to those who do not suffer from diabetes. Type 2 diabetes mellitus patients having albuminuria experience cardiovascular complications; although the precise cause of DN is not known, growth factors, angiotensin II, glomerular hyperfiltration or hyperperfusion, endothelin, and advanced glycation end products (AGEs) are some factors that cause structural alterations in the glomerulus and elevated glomerular capillary pressure [5].

Dipeptidyl peptidase-4 (DPP-4) inhibitors produce defensive mechanisms independently for glucose-lowering actions and blood pressure and are the subject of recent anti-diabetic drugs with renoprotective benefits that have drawn much attention [6]. DPP-4 increases insulin synthesis by acting on the pancreas (beta-cells), their mechanism of action being the prevention of the breakdown of glucagon-like peptides 1 and 2 (GLP-1 and -2) and hence lowering the blood glucose levels. GLP-1 and 2 imitate the action of the glucose-dependent insulinotropic peptide (GIP), promoting insulin production, which is glucose-dependent [7]. Linagliptin, a DPP-4 inhibitor, does the same. Large clinical trials have shown clinically significant results in people with type 2 diabetes mellitus with linagliptin, either by itself or with oral antidiabetic drugs combined, although these trials have shown a lower hypoglycemia risk without weight gain [8-10]. The pooled data (from eight phase III trials) have shown good tolerance to linagliptin in patients with or without renal impairment [11]. This systematic review and meta-analysis (SRMA) were done to synthesize available data to better understand the renoprotective effect of linagliptin in diabetic patients with nephropathy.

## Review

Methodology

Following the Preferred Reporting Items for Systematic Review and Meta-Analyses (PRISMA) criteria [12], the systematic review was carried out and reported. The SRMA was registered with the PROSPERO ID: CRD42023460752.

Data Sources and Search Strategy

Three independent reviewers (PSP, ID, and IM) identified pertinent articles from a systematic literature search of medical electronic databases: Web of Science (WOS), Scopus, and PubMed, to identify all published studies reporting “the reno-protective effect of linagliptin in patients with diabetic nephropathy.” Studies conducted on non-human subjects in languages other than English and without data on the outcomes of interest were not included. Two authors (PSP and ID) separately searched WOS, Scopus, and PubMed for articles published from January 2013 to December 2022 using the keywords linagliptin, diabetic patient, and nephropathy. A third independent reviewer (IM) screened and reviewed any disagreement. The preliminary search generated 397 relevant articles; the duplicates (62) were removed by Rayyan (Rayyan Systems Inc., Cambridge, MA) software, and 335 records remained for review.

Inclusion and Exclusion Criteria

The SRMA included randomized control trials (RCTs), satisfying the inclusion criteria of including type 2 diabetes mellitus with CKD, age 18-80 years, glycated hemoglobin (HbA1c)>6.5%, and treatment with linagliptin for a minimum of three months. If at least two studies reported quantitative data for an outcome, that outcome was included in the meta-analysis. Either numbers or the mean, median, interquartile range, or standard deviation (SD) could be used. We excluded studies reporting end-stage renal disease patients, on dialysis, heart failure, nephrotic syndrome, and cancers.

Study Selection Process

The process of abstract and title screening was done by two reviewers (AM, SKP) independently; applying the inclusion criteria and excluding studies based on the defined exclusion criteria, the reviewers identified 158 articles. Following full-text analysis, five articles were found to be eligible. Initially, all of the identified studies were combined and uploaded into Mendeley (London, UK) software; duplicates were then eliminated. Two independent reviewers checked abstracts and titles against the inclusion criteria (PSD and ID). These two reviewers obtained the full-text versions of the eligible studies and evaluated them. At any point, disagreements during the selection process were settled by another reviewer (IM) independently.

Quality Assessment

The methodological quality was assessed for the included studies using the CONSORT checklist [13]. Other authors (IM, AM, and TJ) discussed and agreed upon any disagreements that arose during the data extraction and quality assessment process. The quality of the articles was adjudged by the Cochrane Risk of Bias assessment tool [14].

Data Extraction

Two authors (SP and TJ) extracted data and assessed quality. The extracted studies were placed into pre-made templates using Microsoft Excel 2016 (Microsoft Corp., Redmond, WA). The results, definitions, and methodological specifics were among the extraction domains. This article presents a narrative summary and tabular descriptions of the results.

Data Analysis 

One author entered the data for the meta-analysis into Microsoft Excel (PSP) and confirmed by another (ID), using the Cochrane RevMan software, version 5.4 (The Cochrane Collaboration, London, UK, 2020). Age, gender, location, number of patients, year of publication, and last name of the author or authors were among the parameters that were extracted from the studies. Heterogeneity was assessed using chi-square, I2, and Tau2. I² values > 50% indicated significant heterogeneity, indicating that the random-effects model should be used. Sensitivity analyses were carried out to evaluate the findings' robustness by removing outliers and low-quality research. Furthermore, the impact of study characteristics on effect sizes was investigated through subgroup analyses and meta-regression. Pooled effect estimates have been reported with a 95% confidence interval (CI) with a p-value < 0.05 (statistically significant). The results have been visually represented as forest plots. To evaluate any potential publication bias, funnel plots were employed using the RevMan software, version 5.4. After searching the literature, 397 studies were found; after eliminating 62 duplicates, 335 articles underwent electronic screening.

**Figure 1:**
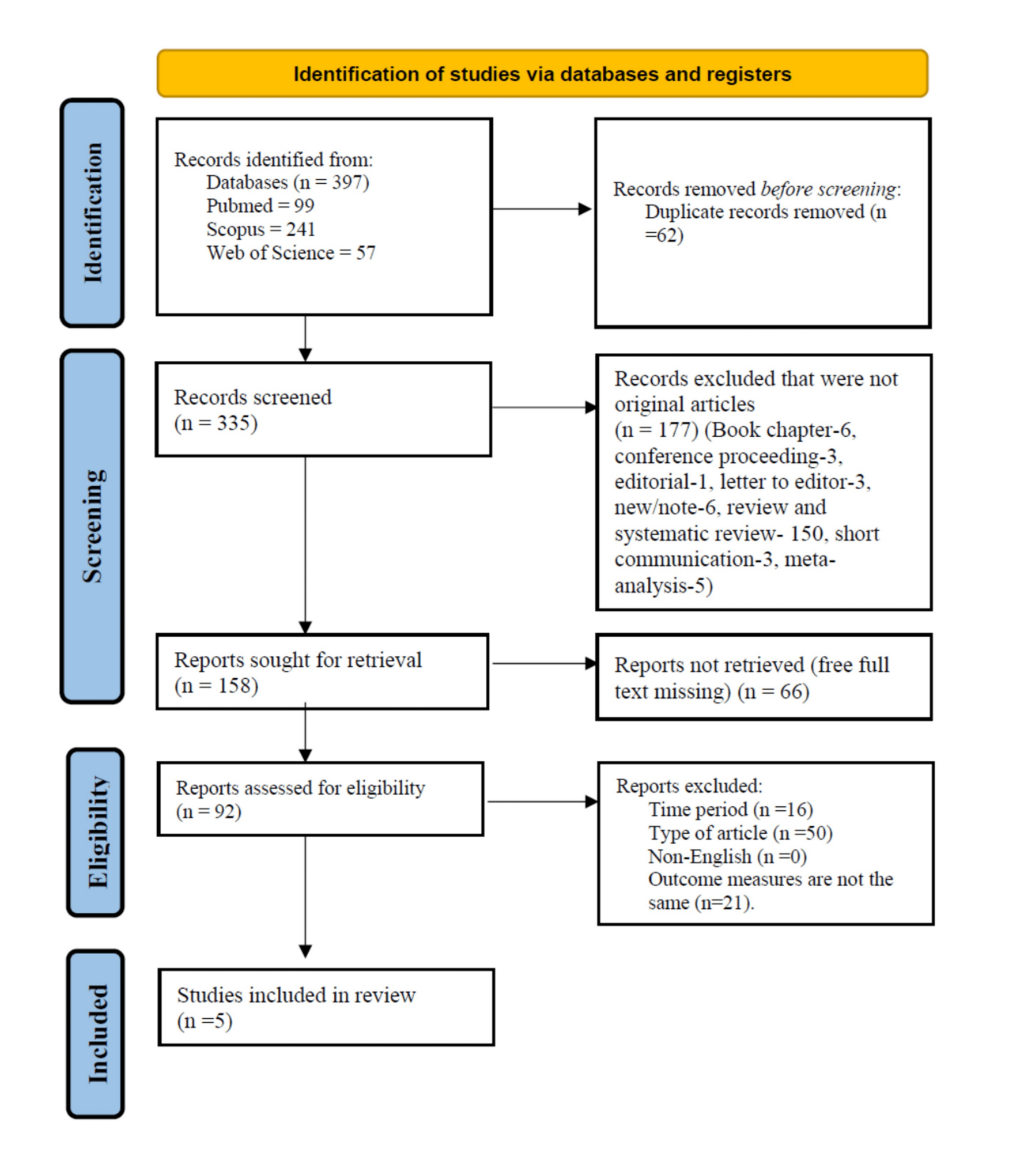
Selection of literature using PRISMA 2020 flow diagram PRISMA: Preferred Reporting Items for Systematic Review and Meta-Analyses

After the titles and abstracts screening, 158 studies were included based on the defined inclusion criteria. For full-text screening, 92 studies were included. Following full-text screening and the collection of outcome measures, five studies were ultimately included for data extraction. The justifications for excluding the studies are mentioned, and the selection of eligible articles is explained in the PRISMA 2020 flow diagram (Figure 1).

Results

Five of the 92 identified studies met the inclusion criteria (Figure 1). These included studies that were published from January 2013 to December 2022 and were from three countries. These five studies included 448 participants, with 188 (42%) male individuals.

Characteristics of Individual Studies

All of the studies were hospital-based studies [15-19] and were carried out between 2013 to 2023. The total sample size was 448 (minimum: 26, maximum: 164) [17,19]. The minimum study duration was 12 weeks [18], and the maximum was 12 months [17,19]. Out of five studies, one study was conducted in Turkey [19], one study was conducted in Japan [17], and three studies were conducted in Iran [15,16,18]. The age group of subjects taken for the studies was>18, >20, and 30-80. In all five studies, both male and female study subjects have been taken.

In the studies by Yagoglu et al. [19], Hirai et al. [17], Moeinzadeh et al. [16], Gharabaghi et al. [18], and Karimifar et al. [15], the participants were taken for drug and placebo were 90 vs 74, 13 vs 13, 62 vs 59, 30 vs 30, and 46 vs 46 respectively. In Table 1 and Table 2, the included studies and the socio-demographics of the participants are described in detail.

**Table 1:** Sociodemographic variables of the studies

Study	Location	Study period	Participants	Age group	Sex
Karimifar et al. [15]	Iran	2023	92	>18	Both
Moeinzadeh et al. [16]	Iran	2021	121	>18	Both
Hirai et al. [17]	Japan	2021	26	>20	Both
Gharabaghi et al. [18]	Iran	2022	60	30-80	Both
Yagoglu te al. [19]	Turkey	2020	164	18-80	Both

**Table 2:** Baseline characteristics of the included studies

Author	Yagoglu et al. [19]	Hirai et al. [17]	Moeinzadeh et al. [16]	Gharabaghi et al. [18]	Karimifar et al. [15]
Year	2020	2021	2021	2022	2023
Place	Turkey	Japan	Iran	Iran	Iran
Number of participants in linagliptin versus placebo	90 vs 74	13 vs 13	62 vs 59	30 vs 30	46 vs 46
Age group	18-80	>20	>18	30-80	>18
Gender (M vs F)	Both (70 vs 94)	Both (14 vs 12)	Both (64 vs 57)	Both (18 vs 42)	Both (22 vs 55)

The mean change in estimated glomerular filtration rate (eGFR) was taken as the primary outcome between the linagliptin group and the placebo group. The mean change was reported by five studies at baseline, by three studies at three months, and by two studies at six months. The eGFR change did not differ significantly between the linagliptin and placebo groups. In only one study, Gharabaghi et al., there is a high mean difference in change in eGFR at baseline and three months [18].

Forest Plot and Risk of Bias

**Figure 2:**
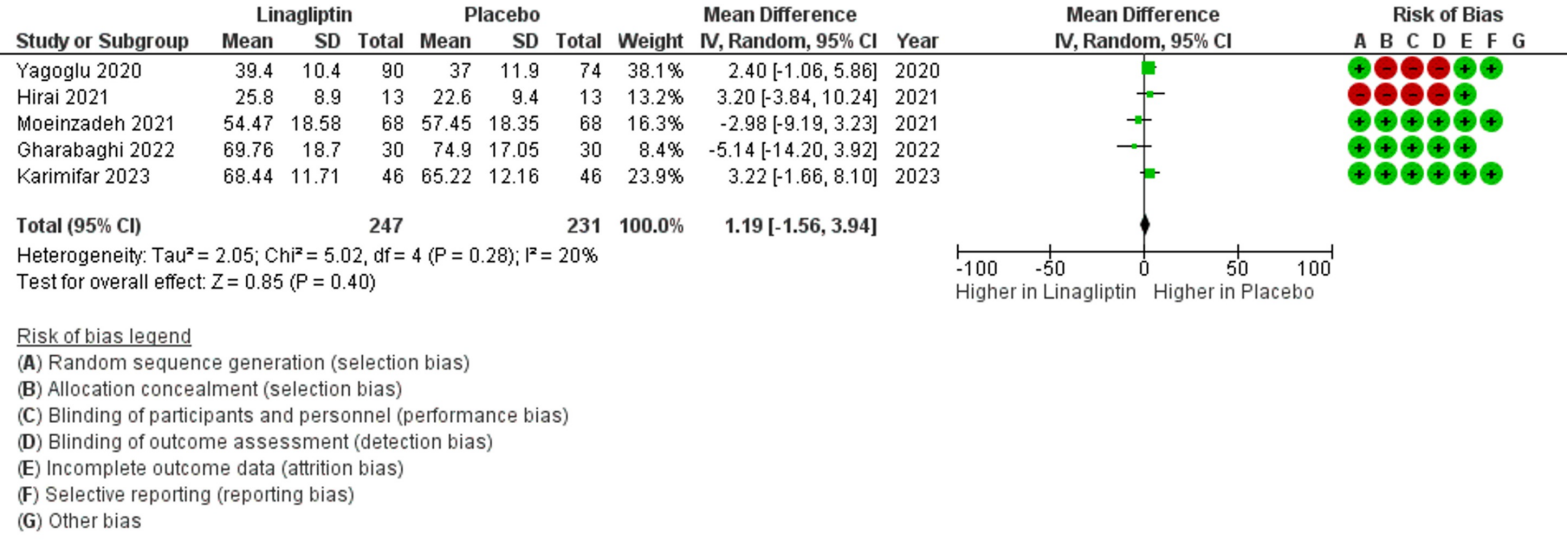
Baseline change in eGFR in the selected five studies Karimifar et al. [15], Moeinzadeh et al. [16], Hirai et al. [17], Gharabaghi et al. [18], Yagoglu et al. [19] eGFR: estimated glomerular filtration rate

The study quality, assessed by the Cochrane Risk of Bias assessment tool, shows that studies were generally of good quality. At baseline, the mean difference was 1.19, which indicates no significant association for linagliptin (p = 0.28) (Figure 2).

**Figure 3:**
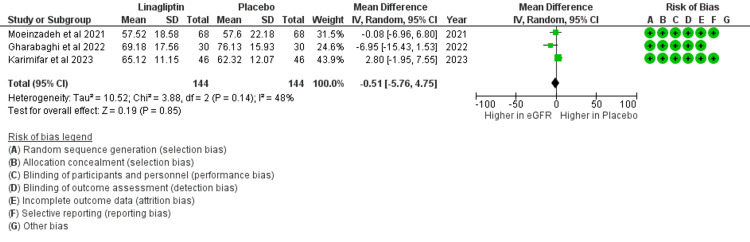
Change in eGFR in the selected three studies after three months of intervention Karimifar et al. [15], Moeinzadeh et al. [16], Gharabaghi et al. [18] eGFR: estimated glomerular filtration rate

The pooled data to assess the mean change in eGFR after three months of intervention in the selected three studies was 0.51, and the p-value was 0.85. Thus, the study results show that the use of linagliptin was not associated with any change in eGFR at three months of intervention (Figure 3).

**Figure 4:**
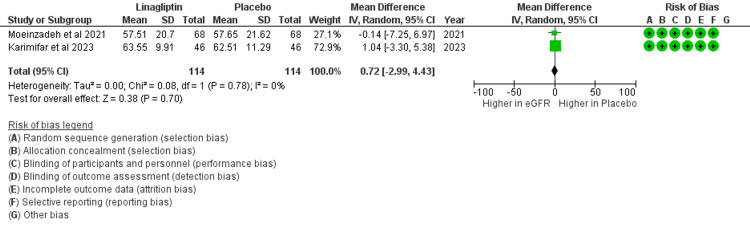
Change in eGFR in the selected two studies after six months of intervention Karimifar et al. [15], Moeinzadeh et al. [16] eGFR: estimated glomerular filtration rate

Similarly, the change in eGFR at six months of intervention had a mean difference of 0.72, showing no association with the renoprotective effect of linagliptin after six months of use (Figure 4).

Publication Bias

**Figure 5:**
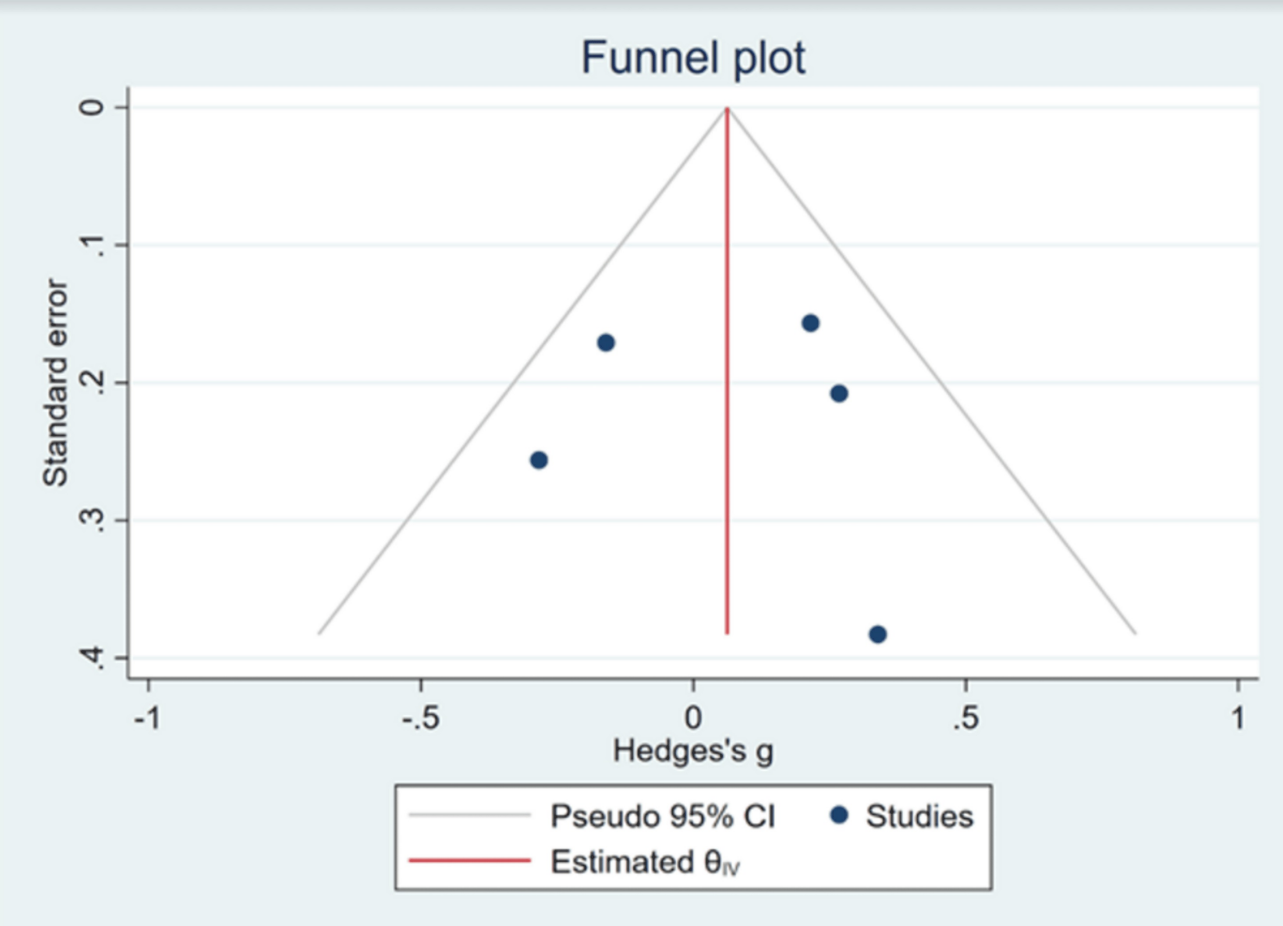
Funnel plot evaluating publication bias

The funnel plot used to evaluate the publication bias related to the meta-analysis (Figure 5) was relatively symmetrical, with data points (studies) scattered evenly around the central vertical line (representing the pooled effect size or estimated θIV). This suggests that there is little evidence of publication bias or small-study effects, as a symmetric distribution indicates that both large and small studies are consistent with the overall meta-analytic effect. The data points are concentrated near the top of the funnel, where the standard errors are smaller, indicating larger studies with more precise estimates. A few points are spread toward the bottom, showing smaller studies with larger standard errors. The funnel plot does not show significant asymmetry, which implies that there is no strong indication of publication bias.

Discussion

An SRMA was done to synthesize available data to better understand linagliptin’s renoprotective effect in diabetic patients with nephropathy.

While various studies evaluated tolerability, safety of DPP-4 inhibitors in patients with impaired kidney function and type 2 diabetes mellitus [20-23], information about the outcomes of clinical trials pertaining to primary kidney disease is currently unavailable [24]. Linagliptin is a suitable candidate to bridge this important gap, is given orally as a single dose to patients with type 2 diabetes mellitus, irrespective of degree of impairment in kidney function, not requiring any adjustment in doses as it has non-renal clearance [25,26].

The mean GFR change was taken as the primary outcome between the groups taking linagliptin and a placebo. The final analysis included five studies with 448 participants. The participants were comprised of 42% males. However, a study by McGill et al., in which they retrospectively analyzed the safety and effectiveness of linagliptin as an adjuvant therapy to sulphonyl urea among patients with uncontrolled type 2 diabetes mellitus and renal impairment, reported a male preponderance (62.6%) [27]. Rosenstock et al. reported 62.9% of males in their CARMELINA RCT [28].

For patients having extremely low GFRs, DPP-4 inhibitors can be used at lower doses [29]; linagliptin has been used (at any dose) in individuals suffering from renal insufficiency [30]. As a result, patients with type 2 diabetes mellitus can be prescribed this medication at a single dose without concern about their kidney function [31,25].

The eGFR change did not differ significantly between the linagliptin and placebo groups. At baseline, the mean difference was 1.19, which indicates no significant association for linagliptin (p = 0.28). The pooled data to assess the change in eGFR after three months of intervention in the selected three studies was 0.51, and the p-value was 0.85. Thus, the study results show that the use of linagliptin was not associated with any change in eGFR at three months of intervention. Similarly, the change in eGFR at six months of intervention had a mean difference of 0.72, showing no association with the renoprotective effect of linagliptin after six months of use.

Comparable results were reported by Groop et al., in their study from Helsinki, Finland, where they assessed the renal and glycemic effects of linagliptin as an adjunct to the standard therapy for type 2 diabetes mellitus patients with albuminuria [32]. They reported that at weeks 6, 12, and 18, there was no discernible difference in the mean change in eGFR between the linagliptin and placebo groups (p = 0.330) [32].

Esaki et al. carried out a comparative study in Japan to examine the effects of DPP-4 inhibitor monotherapy on renal function by retrospectively analyzing the information about patients with type 2 diabetes who were treated with hypoglycemic medications [33]. They, however, reported a significantly higher reduction of eGFR rate, from baseline to three (p = 0.034) and twelve months (p = 0.020), among patients treated with DPP-4 inhibitors compared to the untreated group. However, the eGFR reduction rate was not significant from baseline to six months (p = 0.052), similar to the present study [33].

DPP-4 acts on the proximal tubular cells of the non-diseased kidney of humans, acting on the apical brush border of these cells [34,35]. Interestingly, it was found that when people developed CKD, DPP-4 was also expressed in the glomeruli, indicating the presence of local adaptive mechanisms [36].

To assess kidney function, doubling serum creatinine levels and the associated eGFR decline of greater than 57% are hard endpoints; nevertheless, long-term monitoring is required [37]. Thus, we recommend a randomized controlled trial of a longer duration to explore the renoprotective action of linagliptin.

Limitation

The final analysis included studies that explored the renoprotective role of linagliptin for a shorter duration. A relatively small number of studies (five data points) restricts the ability to draw definitive conclusions regarding the presence or absence of bias. Random and fixed-effect modeling could not be done.

## Conclusions

Despite preclinical and observational data suggesting potential renoprotective properties, linagliptin did not demonstrate a significant effect on slowing kidney disease progression in this analysis. For diabetes patients with chronic renal disease, linagliptin may be a helpful renoprotective treatment choice. Extensive, long-term research can be undertaken, which can help generate robust scientific evidence and help in the practice of evidence-based medicine.
